# Phenotypic characterization of inducible and constitutive clindamycin resistance in clinical *Staphylococcus aureus* isolates

**DOI:** 10.1590/S1678-9946202668061

**Published:** 2026-09-18

**Authors:** Manal Rossi, Moussa Benboubker, Salim Belchkar, Ghita Yahyaoui, Mustapha Mahmoud, Bouchra Oumokhtar

**Affiliations:** 1 Université Sidi Mohamed Ben Abdellah de Fès, Faculté de Médecine, de Pharmacie et de Médecine Dentaire, Laboratoire de Pathologie Humaine, Biomédecine et Environnement, Fès, Morocco; 2 Université Sidi Mohamed Ben Abdellah de Fès, Faculté de Médecine, de Pharmacie et de Médecine Dentaire, Laboratoire d’Épidémiologie et Recherche en Sciences de la Santé, Fès, Morocco; 3 Université Sidi Mohamed Ben Abdellah de Fès, Faculté de Médecine, de Pharmacie et de Médecine Dentaire, Laboratoire de Recherche Biomédicale et Translationnelle, Fès, Morocco

**Keywords:** MLS_B_ resistance, Staphylococcus aureus, MRSA, D-test, Antimicrobial susceptibility

## Abstract

*Staphylococcus aureus* is a major human pathogen causing infections ranging from mild skin infections to life-threatening systemic conditions. Clindamycin has long been used to treat serious *Staphylococcus aureus* infections due to its pharmacokinetic profile and ability to inhibit toxin production. However, inducible resistance to clindamycin via erythromycin ribosomal methylases encoded by *erm* genes poses a significant clinical challenge; it often leads to treatment failure and remains undetected by routine antimicrobial susceptibility testing. This cross-sectional study analyzed 120 non-duplicate clinical *Staphylococcus aureus* isolates from the University Hospital Center of Fez, Morocco. Identification followed standard microbiological methods, and susceptibility testing adhered to the European Committee on Antimicrobial Susceptibility Testing (EUCAST/CA-SFM) 2024 guidelines. Inducible clindamycin resistance was assessed using the D-test, while methicillin resistance was determined by the cefoxitin disk diffusion method. Of the 120 isolates, 50 (41.67%) were resistant to erythromycin. Constitutive macrolide-lincosamide-streptogramin B (MLS_B_) resistance was detected in 31 isolates (25.83%), inducible MLS_B_ (iMLS_B_) resistance in 5 isolates (4.17%), and the macrolide-streptogramin B (MS) phenotype in 14 isolates (11.66%). All isolates with inducible clindamycin resistance or the MS phenotype were methicillin-susceptible *Staphylococcus aureus* (MSSA), while constitutive MLS_B_ resistance was more frequent among methicillin-resistant *Staphylococcus aureus* (MRSA). Inducible clindamycin resistance, though less prevalent, remains clinically significant and can be readily detected using the D-test. Routine use of the D-test is essential to guide appropriate antimicrobial therapy and prevent clindamycin treatment failure.

## INTRODUCTION

*Staphylococcus aureus* is considered one of the main and most common pathogens in clinical medicine. It is known for acquiring antimicrobial resistance shortly after the introduction of new antibiotics, causing a range of infections from simple skin abscesses to potentially fatal systemic infections such as sepsis and endocarditis^1^. The clinical control of these infections depends strongly on the successful use of antibiotics. Among these, clindamycin is of particular importance because of its enhanced tissue penetration, improved oral bioavailability, and unique capacity to inhibit the production of bacterial toxins, which are important virulence factors in *S. aureus* ^2^. However, the increasing global incidence of clindamycin resistance poses a severe risk to therapeutic effectiveness and patient outcomes^3^.

Resistance to macrolide, lincosamide, and streptogramin B (MLS_B_) antibiotics in *S. aureus* involves three mechanisms: 1) target site modification by erythromycin ribosomal methylases encoded by *erm* genes, which methylate the 23S rRNA A2058 residue; 2) active drug efflux mediated by the *msrA* gene; and 3) enzymatic inactivation of the antibiotic^4^. The *erm*-mediated methylation produces cross-resistance within the MLS_B_ class and can be expressed as either constitutive MLS_B_ (cMLS_B_) or inducible MLS_B_ (iMLS_B_) phenotypes; while cMLS_B_ resistance is easily detected, iMLS_B_ remains “hidden” during routine testing, as isolates appear susceptible until exposed to an inducing agent such as erythromycin^4^ ^-^ ^6^. This iMLS_B_ phenotype heightens the risk of treatment failure, disease recurrence, and spread of resistant strains in healthcare settings^7^.

To overcome this diagnostic limitation, the D-test was developed as an inexpensive and easy phenotypic assay that places erythromycin and clindamycin disks in proximity to each other on agar plates inoculated with an *S. aureus* isolate, based on the principle of induction: a flattening of the zone of inhibition around the clindamycin disk, creating a characteristic D-shape, is taken as an indication of inducible resistance^6^ ^,^ ^8^. Although this laboratory procedure is simple, it is a crucial clinical tool for guiding appropriate antibiotic therapy^9^.

Current guidelines recommend restricting the use of clindamycin to infections with confirmed susceptibility, thereby protecting patients from the risk of treatment failure and adverse outcomes^10^.

Given the increasing frequency of antimicrobial resistance, particularly in resource-limited nations like Morocco, it is important to define both the prevalence and type of inducible clindamycin resistance among *S. aureus* isolates. Therefore, this study aims to bridge this knowledge gap by determining the prevalence and phenotypic distribution of MLS_B_ resistance, with a particular focus on inducible clindamycin resistance, and by highlighting the paramount importance of the D-test in modern clinical microbiological practice and its relevance in the fight against antibiotic resistance. The study was conducted at the University Hospital Center of Fez. 

To the best of our knowledge, published data on the routine evaluation of inducible clindamycin resistance using the D-test in clinical *S. aureus* isolates remain limited in our setting, highlighting the importance of this study for regional and national epidemiological surveillance and clinical practice.

### Ethics

Ethical approval has been granted by the ethical committee of CHU Hassan II University Hospital, Maroc, process Nº 05/24, date of approval 2024. Patient data were anonymized prior to analysis, and no personal identifiers were retained in the study dataset.

## MATERIALS AND METHODS

This cross-sectional study was conducted at the Hassan II University Hospital Center (CHU) of Fez, Morocco, a tertiary referral center serving the population across the Fez-Meknes region, and included 120 clinical isolates of *S. aureus* recovered from various specimens such as pus, swabs, aspirates, blood, sterile fluids, catheters, and urine from patients with active infections. Non-duplicate isolates were defined as unique clinical isolates, with each isolate originating from a distinct patient, confirmed by reviewing patient records using the unique hospital identification number, to avoid inclusion of repeat cultures from the same individual. Isolates were collected over a six-month period during 2024. 

Identification of *S. aureus* was carried out using standard microbiological techniques applied to all 120 isolates. Colony morphology was examined on Mannitol Salt Agar (MSA), a selective and differential medium on which *S. aureus* characteristically produces golden-yellow colonies, due to the fermentation of mannitol, which leads to acidic conditions and a color change in the phenol red pH indicator. Gram staining confirmed Gram-positive cocci arranged in clusters. Catalase testing yielded positive results for all isolates, differentiating them from *Streptococcus* spp. Coagulase testing was positive in all isolates, confirming species identity as *S. aureus*. DNase testing was also performed and was positive for all isolates.

Antimicrobial susceptibility testing was performed using the Kirby-Bauer disk diffusion technique on Mueller Hinton Agar (MHA), as recommended by the EUCAST/CA-SFM 2024 guidelines^10^. For each isolate, a 0.5 McFarland standard suspension was inoculated onto MHA plates.

**Figure 1:**
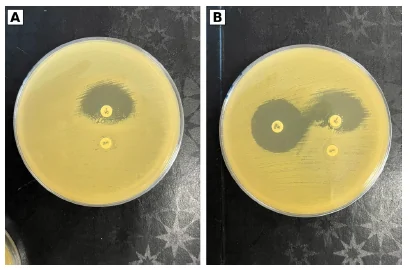
Phenotypic detection of inducible clindamycin resistance (D-test) and methicillin susceptibility: (A) Representative image of a positive D-test result. The flattening of the zone of inhibition around the clindamycin disk (2 µg) adjacent to the erythromycin disk (15 µg) creates the characteristic D-shape, indicating the inducible MLS_B_ phenotype; (B) Detection of iMLS_B_ resistance in a methicillin-susceptible *S. aureus* isolate. This panel shows a positive D-test alongside susceptibility to cefoxitin (30 µg), confirming the isolate is not MRSA.

Inducible clindamycin resistance was detected using the D-test, in which an erythromycin disk (15 µg) and a clindamycin disk (2 µg) were placed 15-20 mm apart, and the plates were incubated at 35 ± 2 °C for 18-24 h. After incubation, a flattened D-shaped zone of inhibition adjacent to the clindamycin disk was interpreted as a positive D-test (Figure 1A)^10^. 

Methicillin resistance was simultaneously screened using a cefoxitin disk (30 µg); isolates were then classified as methicillin-susceptible *S. aureus* (MSSA) or methicillin-resistant *S. aureus* (MRSA).

Quality control was performed with *S. aureus* ATCC 25923^10^. Based on the D-test results, isolates were categorized into three phenotypic groups: Constitutive MLS_B_ phenotype - resistant to both erythromycin and clindamycin, reflecting phenotypic constitutive MLS_B_ resistance. This classification is based on phenotypic criteria and does not exclude underlying inducible genetic mechanisms at the molecular level. Inducible MLS_B_ phenotype - erythromycin resistant, clindamycin susceptible, with a flattening of the zone of inhibition toward the erythromycin disk producing a D-shaped blunting (D-test positive), which suggests a resistance phenotype due to the expression of the *erm* gene. And MS phenotype - erythromycin resistant but susceptible to clindamycin, with a circular zone of inhibition around clindamycin (D-test negative), suggesting resistance due to an *msrA*-encoded active efflux pump mechanism^10^.

Data were recorded and organized using Microsoft Excel. Statistical analysis was performed using IBM SPSS Statistics software. Descriptive statistics were used to summarize the distribution of antimicrobial resistance phenotypes, with results expressed as absolute frequencies and percentages. The association between methicillin resistance and clindamycin resistance phenotypes was assessed using the chi-square test, with odds ratios (OR) and 95% confidence intervals (CI) calculated where appropriate. A p-value of less than 0.05 was considered statistically significant.

## RESULTS

A total of 120 clinical *S. aureus* strains were analyzed in the study. The isolates were recovered from various specimen types, with purulent samples representing the most frequent source, followed by blood cultures (hemocultures) and other specimen types including lumbar puncture samples. Based on cefoxitin susceptibility testing, 27 isolates (22.5%) were identified as methicillin-resistant *S. aureus* (MRSA) and 93 isolates (77.5%) were methicillin-susceptible *S. aureus* (MSSA).

Among all isolates tested, 50 (41.67%) were resistant to erythromycin, and 70 (58.33%) were susceptible to both erythromycin and clindamycin. Among the 50 isolates resistant to erythromycin, three phenotypic patterns were identified based on clindamycin susceptibility and D-test results for inducible clindamycin resistance.

In this regard, 31 isolates (25.83%) exhibited constitutive MLS_B_ resistance (cMLS_B_ phenotype) as they were resistant to both erythromycin and clindamycin. Five isolates (4.17%) showed inducible clindamycin resistance (iMLS_B_ phenotype), whereas 14 isolates (11.66%) showed the MS phenotype, characterized by erythromycin resistance and clindamycin susceptibility along with a negative D-test result (Table 1). Among the five isolates exhibiting inducible MLS_B_ resistance (iMLS_B_, D-test positive), two were isolated from blood cultures, two from purulent samples, and one from a catheter tip, all of which were methicillin-susceptible S. *aureus* (MSSA). 

**Table 1:** Susceptibility to erythromycin (ERY) and clindamycin (CL) among all S. aureus isolates.

Susceptibility pattern (Phenotype)	Number of isolates	Percentage
ERY-S, CL-S	70	58.33 %
ERY-R, CL-R (Constitutive MLS_B_)	31	25.83 %
ERY-R, CL-S (D-test positive, iMLS_B)_	5	4.17 %
ERY-R, CL-S (D-test negative, MS)	14	11.66 %
Total	120	100

ERY = Erythromycin; CL = Clindamycin; S = Susceptible; R = Resistant; Constitutive MLS_B =_ Constitutive MLS_B_ phenotype; iMLS_B =_ Inducible MLS_B_ phenotype; MS = MS phenotype.

Analysis of resistance phenotypes in relation to methicillin susceptibility, as shown in Table 2, revealed distinct differences between MRSA and MSSA isolates. All five isolates exhibiting inducible MLS_B_ resistance were MSSA and none were MRSA (Figure 1B). In contrast, among the 31 isolates with constitutive MLS_B_ resistance, 19 were MRSA while 12 were MSSA. Regarding the MS phenotype, 14 isolates were identified, the majority of which were MSSA (12 isolates), whereas only two were MRSA.

**Table 2:** Association of clindamycin resistance with methicillin resistance.

Clindamycin resistance	Methicillin resistance		Total (%) (n=120)
	MRSA	MSSA	
ERY-S, CL-S	6	64	58.33 %
ERY-R, CL-R (Constitutive MLS_B_)	19	12	25.83 %
ERY-R, CL-S (D-test positive, iMLS_B_)	0	5	4.17 %
ERY-R, CL-S (D-test negative, MS)	2	12	11.66 %

ERY = Erythromycin; CL = Clindamycin; S = Susceptible; R = Resistant; Constitutive MLS_B =_ Constitutive MLS_B_ phenotype; iMLS_B_= Inducible MLSB phenotype; MS = MS phenotype; MRSA = Methicillin-resistant *Staphylococcus aureus*; MSSA = Methicillin-susceptible *Staphylococcus aureus*.

A statistically significant association was observed between methicillin resistance and constitutive clindamycin resistance. MRSA isolates were significantly more likely to exhibit the cMLS_B_ phenotype compared to MSSA isolates (OR = 16.03; 95% CI: 5.77-44.6; p < 0.0001).

The antimicrobial susceptibility profile of the *S. aureus* isolates demonstrated high susceptibility to norfloxacin (95%), gentamicin (87.5%), and rifampicin (85.83%). In contrast, erythromycin resistance was observed in 50 isolates (41.67%).

True clindamycin resistance-defined as isolates exhibiting either constitutive MLS_B_ (cMLS_B_) or inducible MLS_B_ (iMLS_B_) phenotypes, both of which are associated with clinical treatment failure-was identified in 36 isolates (30%), in accordance with EUCAST/CA-SFM 2024 guidelines (Table 3).

**Table 3:** Distribution of susceptibility and resistance among S. aureus isolates to selected antibiotics according to EUCAST/CA-SFM 2024 guidelines.

Class of antibiotics	Antibiotic	Susceptibility (%)	Resistance (%)
Macrolides	Erythromycin (15 ug)	58.33	41.67
Lincosamides	Clindamycin (2 ug)	70	30
Aminoglycosides	Gentamicin (10 ug)	87.5	12.5
Cephalosporins	Cefoxitin (30 ug)	77.5	22.5
Fusidane	Fusidic acid (10 ug)	77.5	22.5
Fluoroquinolones	Norfloxacin (10 ug)	95	5
Rifamycins	Rifampicin (5 ug)	85.83	14.17

## DISCUSSION

This study offers key insights into the resistance patterns of *S. aureus* within the CHU of Fez, in Morocco, with special emphasis on clindamycin resistance mechanisms and their phenotypic variation. Among the 120 *S. aureus* isolates, 50 (41.67%) were erythromycin resistant, indicating a significant resistance rate and highlighting the importance of prudent antibiotic use in clinical settings^11^. 

The resistance phenotypes were distributed as follows: 31 isolates (25.83%) exhibited constitutive MLS_B_ resistance, 5 (4.17%) showed inducible resistance (iMLS_B_, D-test positive), and 14 (11.66%) isolates were found to have the MS phenotype (erythromycin-resistant, clindamycin-susceptible, D-test negative). 

An important observation was the distribution of resistance phenotypes in relation to methicillin resistance status: while the constitutive MLS_B_ (cMLS_B_) phenotype was predominantly associated with MRSA strains, the inducible (iMLS_B_) and efflux-mediated (MS) phenotypes were exclusively or primarily identified among MSSA isolates. This strong statistical correlation (OR = 16.03; p < 0.0001) suggests that in our clinical setting, methicillin resistance often co-segregates with phenotypic constitutive MLS_B_ resistance, likely reflecting high-level *erm* gene-mediated methylation. However, as molecular characterization was not performed, the precise genetic basis of this phenotypic pattern-including the potential contribution of inducible *erm* gene variants that phenotypically mimic constitutive resistance-cannot be definitively established. This is consistent with the global characterization of MRSA as a multidrug-resistant entity that frequently leaves clinicians with few viable therapeutic alternatives.

The observation that all iMLS_B_ isolates were methicillin-susceptible suggests a unique local epidemiology. This finding implies that the genetic determinants for inducible resistance may be circulating within distinct, methicillin-susceptible clonal lineages in the Fez region. This phenotypic segregation may be further explained by the specific distribution of *erm* gene classes. Previous studies have reported that the *ermC* gene is frequently localized on small plasmids and is often found in MSSA strains, whereas *ermA* gene is typically integrated into the chromosome within the Tn554 transposon, a configuration more common in MRSA^12^. 

Although molecular characterization was not performed in this study, these observed patterns suggest that the local prevalence of specific genetic alleles, such as the *ermC* gene, could be driving the inducible resistance seen exclusively in our MSSA isolates^12^.

Furthermore, the higher prevalence of the MS phenotype among MSSA (12 isolates) versus MRSA (2 isolates) indicates that active efflux mechanisms, likely encoded by the *msrA* gene, represent a significant alternative pathway for macrolide resistance in non-MRSA strains. These findings underscore the complexity of staphylococcal resistance; they demonstrate that even methicillin-susceptible strains can harbor “hidden” resistance mechanisms that are undetectable by standard susceptibility panels. Consequently, reliance on the D-test is not merely supplementary but essential for preventing the inadvertent use of clindamycin in cases where therapeutic failure is genetically predetermined.

Notably, 70 (58.33%) of the isolates remained fully susceptible to both erythromycin and clindamycin, which indicates that these antibiotics may still hold promise as treatment options in a proportion of clinical cases. The fact that inducible resistance was only observed among MSSA strains may reflect differences in the distribution of resistance determinants between MSSA and MRSA populations. The overall antimicrobial susceptibility profile of the isolates revealed encouraging activity for several non-macrolide agents. Gentamicin demonstrated high susceptibility (87.5%), consistent with its established role as an adjunctive agent in the management of serious *S. aureus* infections, including combination regimens for endocarditis and bacteremia². Norfloxacin also showed high susceptibility (95%), suggesting preserved fluoroquinolone activity in our clinical setting. These findings suggest that, in our clinical setting, gentamicin may represent a valuable adjunctive therapeutic option, provided susceptibility is confirmed, and appropriate combination regimens are followed. Importantly, given that the majority of isolates-including all five iMLS_B_ strains-were MSSA, and that 58.33% of all isolates remained fully susceptible to both erythromycin and clindamycin, the overall therapeutic landscape remains relatively preserved, with multiple viable antibiotic options available for most clinical cases encountered in our setting.

However, further molecular studies would be required to confirm the underlying genetic mechanisms. These findings highlight the complexity of resistance patterns in clinical *S. aureus* isolates and emphasize the importance of accurate phenotypic detection methods when evaluating clindamycin susceptibility^12^.

Across diverse geographic settings, the prevalence of methicillin-resistant *S. aureus* (MRSA) and MLS_B_ resistance phenotypes show considerable variability. In a systematic review of African studies, Assefa^13^ reported an overall prevalence of inducible MLS_B_ resistance of 19.8% among 3,064 *S. aureus* isolates, with reported rates ranging from 2.9% to 44%, and a higher prevalence among MRSA strains. In the same review, Egypt was identified as one of the countries with the highest prevalence of inducible resistance, reaching 44% of isolates and 77.8% among MRSA strains^13^. In Jordan, Jarajreh *et al*.^14^ analyzed 126 staphylococcal isolates and identified 71 *S. aureus* strains, of which 55 (77.5%) were MRSA. Among erythromycin-resistant MRSA isolates, inducible MLS_B_ resistance was detected in 33 isolates (76.7%), whereas constitutive resistance accounted for 18.6%. In India, Gupta *et al*.^15^ reported that among 190 clinical *S. aureus* isolates, 10% exhibited inducible MLS_B_ resistance and 9% constitutive resistance, with both phenotypes more frequently observed among MRSA isolates. Similarly, Banik *et al*.^16^ reported that among 243 clinical *S. aureus* isolates in India, 39% were erythromycin resistant, with inducible and constitutive MLS_B_ resistance detected in 10.7% and 16.9% of isolates, respectively, and both phenotypes occurring more frequently among MRSA isolates.

In Iran, Saderi *et al*.^17^ reported that, out of 211 *S. aureus* isolates, erythromycin resistance was expressed in 88.6% of MRSA isolates and clindamycin resistance in 52.3%, with inducible clindamycin resistance detected in 20.5% of MRSA isolates.

A comparative overview of iMLSB resistance rates and their association with methicillin susceptibility across geographic settings is summarized in Table 4.

**Table 4:** Comparison of staphylococcal iMLS_B_ phenotypes and methicillin susceptibility patterns across different regions.

Region (Study)	Sample size (n)	iMLS_B_ prevalence	Predominant Status (iMLS_B_)
Fez, Morocco (This study)	120	4.17%	100% MSSA
Africa (Assefa^13^)	3,064	19.8%	Higher among MRSA
India (Gupta *et al.* ^15^)	190	10%	More frequent among MRSA
India (Banik *et al*.^16^)	243	10.7%	More frequent among MRSA
Jordan (Jarajreh *et al*.^14^)	71	76.7%*	Studied among MRSA only
Iran (Saderi *et al.* ^17^)	211	20.5%**	Studied among MRSA only

*Percentage reported specifically among erythromycin-resistant S. aureus isolates; **Percentage reported specifically within the MRSA isolates.

Collectively, these findings indicate that MLS_B_ resistance in *S. aureus* exhibits important geographic variability, despite its frequent association with methicillin-resistant strains (MRSA). This heterogeneity likely reflects differences in antimicrobial use and the circulation of distinct resistant clones across settings, underscoring the need for locally generated resistance data to guide empirical therapy. In parallel, molecular investigations have shown that these phenotypes are predominantly mediated by methylase-encoding *erm* genes and, to a lesser extent, by efflux mechanisms involving the *msrA* gene, as demonstrated in previously mentioned studies^16^ ^,^ ^17^. Overall, these findings highlight that combining phenotypic methods with molecular approaches is essential for accurately characterizing resistance patterns and strengthening antimicrobial stewardship strategies.

### Limitations of the study

Several limitations should be acknowledged when interpreting these findings. The sample size of 120 isolates from a single tertiary center may limit the generalizability of results at the regional or national level. The six-month collection period may not capture temporal variations in resistance profiles related to seasonal changes or emerging resistance mechanisms. Additionally, the exclusive use of phenotypic methods, without molecular characterization or MIC confirmation, limits the ability to define the precise genetic basis of the observed resistance patterns. Future studies incorporating larger sample sizes, multicenter designs, longer collection periods, and molecular approaches would provide a more comprehensive understanding of clindamycin resistance dynamics and further strengthen antimicrobial stewardship strategies.

## CONCLUSION

This study characterizes the phenotypic distribution of MLS_B_ resistance among 120 clinical *S. aureus* isolates at the CHU of Fez, Morocco. Erythromycin resistance was detected in 41.67% of isolates, with true clindamycin resistance-encompassing both constitutive (cMLS_B_, 25.83%) and inducible (iMLS_B_, 4.17%) phenotypes-identified in 30% of isolates. A statistically significant association between methicillin resistance and the cMLS_B_ phenotype was observed (OR = 16.03; p < 0.0001), while iMLS_B_ and MS phenotypes were exclusively or predominantly found among MSSA isolates, suggesting distinct epidemiological dynamics in our setting. These findings underscore the clinical importance of routine D-test implementation, as inducible clindamycin resistance remains undetectable by standard susceptibility testing alone and carries significant risk of treatment failure. Locally generated resistance data of this kind are essential to guide empirical therapy and strengthen antimicrobial stewardship strategies in resource-limited settings.

## Data Availability

The anonymized dataset generated during this study is available from the corresponding author upon reasonable request.

## References

[1] Tong SY, Davis ES, Eichenberger E, Holland TL, Fowler VG (2015). Staphylococcus aureus infections epidemiology, pathophysiology, clinical manifestations, and management. Clin Microbiol Rev.

[2] Liu C, Bayer A, Cosgrove AS, Daum RS, Fridkin SK, Gorwitz RJ (2011). Clinical practice guidelines for the management of methicillin-resistant Staphylococcus aureus infections in adults and children 2011 update by the Infectious Diseases Society of America. Clin Infect Dis.

[3] Lowy FD (2003). Antimicrobial resistance the example of Staphylococcus aureus. J Clin Invest.

[4] Leclercq R (2002). Mechanisms of resistance to macrolides and lincosamides nature of the resistance elements and their clinical implications. Clin Infect Dis.

[5] Weisblum B (1995). Erythromycin resistance by ribosome methylation. Antimicrob Agents Chemother.

[6] Fiebelkorn KR, Crawford SA, McElmeel ML, Jorgensen JH (2003). Practical disk diffusion method for detection of inducible clindamycin resistance in Staphylococcus aureus and coagulase-negative staphylococci. J Clin Microbiol.

[7] Steward CD, Raney PM, Morrell AK, Williams PP, McDougal LK, Jevitt L (2005). Testing for induction of clindamycin resistance in erythromycin-resistant isolates of Staphylococcus aureus. J Clin Microbiol.

[8] Deotale V, Mendiratta DK, Raut U, Narang P (2010). Inducible clindamycin resistance in Staphylococcus aureus isolated from clinical samples. Indian J Med Microbiol.

[9] Goyal R, Singh NP, Manchanda V, Mathur M (2004). Detection of clindamycin susceptibility in macrolide resistant phenotypes of Staphylococcus aureus. Indian J Med Microbiol.

[10] Société Française de Microbiologie.European Committee on Antimicrobial Susceptibility Testing (2024). Comité de l'Antibiogramme de la Société Francaise de Microbiologie: recommandations 2024, V.1.0 Juin.

[11] Majhi S, Dash M, Mohapatra D, Mohapatra A, Chayani N (2016). Detection of inducible and constitutive clindamycin resistance among Staphylococcus aureus isolates in a tertiary care hospital, Eastern India. Avicenna J Med.

[12] Lina G, Quaglia A, Reverdy ME, Leclercq R, Vandenesch F, Etienne J (1999). Distribution of genes encoding resistance to macrolides, lincosamides, and streptogramins among staphylococci. Antimicrob Agents Chemother.

[13] Assefa M (2022). Inducible clindamycin-resistant Staphylococcus aureus strains in Africa a systematic review. Int J Microbiol.

[14] Jarajreh D, Aqel A, Alzoubi H, Al-Zereini W (2017). Prevalence of inducible clindamycin resistance in MRSA the first study in Jordan. J Infect Dev Ctries.

[15] Gupta V, Datta P, Rani H, Chander J (2009). Inducible clindamycin resistance in Staphylococcus aureus a study from North India. J Postgrad Med.

[16] Banik A, Khyriem AB, Gurung J, Lyngdoh VW (2015). Inducible and constitutive clindamycin resistance in Staphylococcus aureus in a northeastern Indian tertiary care hospital. J Infect Dev Ctries.

[17] Saderi H, Owlia P, Eslami M (2009). Prevalence of macrolide-lincosamide-streptogramin B (MLSB) resistance in Staphylococcus aureus isolated from patients in Tehran, Iran. Iran J Pathol.

